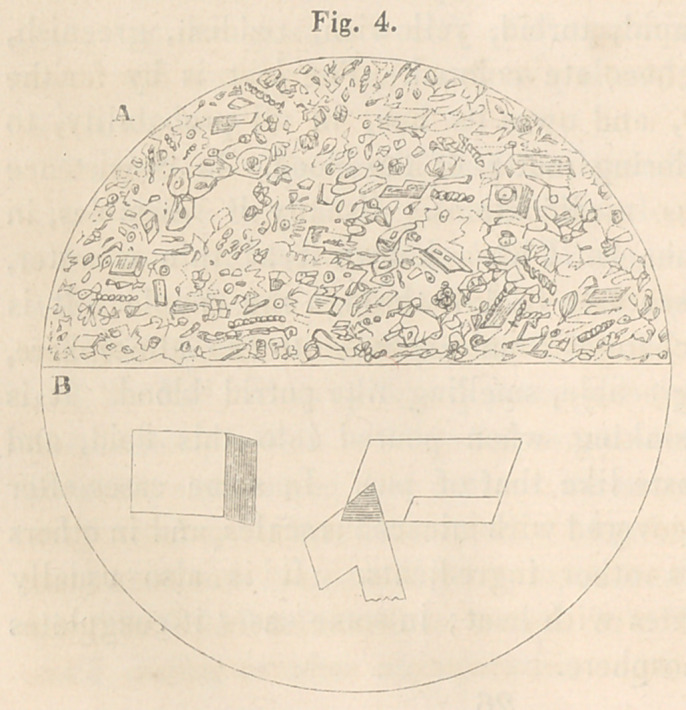# Hydrocele of the Neck

**Published:** 1850-05

**Authors:** Thomas D. Mütter

**Affiliations:** Professor of Surgery in Jefferson Medical College


### MEDICAL EXAMINER,

AND

### RECORD OF MEDICAL SCIENCE.

NEW SERIES.—NO. LXV.—MAY, 1850.

### ORIGINAL COMMUNICATIONS.

*Hydrocele of the Neck.* By Thomas D. Mütter, M. D., Professor of Surgery in Jefferson Medical College. *With cases and illustrations.* (*Clinic of Jefferson Medical College.*)

Case 1.—On the 1st of November, 1848, Mrs. G. was delivered of a female child, well formed, with the exception of several tumours of considerable size, which covered the chin, anterior part of the neck, and the upper part of the sternum. From this period they continued to increase in size, and to extend more and more into the surrounding parts. (See fig. 1.)

About a week after birth, the child was sent by Dr. Bibighaus, the physician of the mother, to the clinic of the Jefferson College, where Prof. Mütter diagnosed the case. The fluid was observed to be contained in several sacs, of varied size, and each apparently independent of the others. It was finally resolved to draw off the contents of one or more of the sacs from time to time, as occasion might demand, in the faint hope of being thus enabled to preserve life until increased age and vigor would give greater chance of success to the employment of some such remedy as the seton.

To verify the diagnosis, acupuncture was employed, and during the same day nearly half an ounce of straw-colored fluid was forced out drop by drop through the aperture thus made. The ease which this gave clearly pointed out the relief to be obtained from operating, and accordingly on the 23d, when the symptoms grew rather urgent, Dr. Mütter drew off three and a half ounces of dark, amber-hued serum, from the upper left side tumor. Almost immediately, respiration became easier, and the slight cough disappeared.

I carried away the serum to watch and examine it more closely. Eight hours after removal, a dense coagulum nearly filled the bottle, and the fluid had darkened very much. Twenty-four hours after, the coagulum had shrunk, expelling a good deal of contained fluid from its substance. Dr. Leidy, who examined it at Dr. Mütter's request, ascertained that it consisted of serum, with few or no blood discs.

On the 25th, the fluid had again returned, and by the 27th had distended the sac to its former size ; the general symptoms keeping pace with its growth. On the 30th, the trocar was again resorted to, in order to relieve the sufferer, and three ounces of serum withdrawn, the same happy effects as before following the operation.

No other resort to the trocar was made until the 13th of December, when three ounces of a darker serum were removed from the lower cyst of the left side. From this time I continued to see the patient constantly, and the trocar was used as often as occasion demanded.

*Jan. 14th.* I found the child much thinner, and laboring under an oppressing cough, which the mother called whooping cough, and which sounded not a little like it. On the 21st, although the debility was marked, the cough had decreased, and the child seemed but little worse than usual. Dr. Mütter resolved to operate again on the following day, but during the night she began to sink rapidly, and about three, A. M., died with scarcely a struggle, and before any physician could be called to her aid.

Thirty-six hours after death, Dr. I. V. Paterson opened the body in the presence of Dr. Mütter, Dr. Bibighaus, Mr. Zimmerman, and myself. An incision was carried from the chin to the middle of the sternum, and the closely adherent and hardened skin dissected from the walls of the sacs. Three large and two small sacs were now seen, running up as far as the pterygoid processes of the sphenoid bone, lying on the anterior part of the spine, and one of them covering the sternal end of the left clavicle and the sternum itself. Numbers of smaller cysts lay scattered along the trachea and through the cellular tissue of the neck, but each was distinct from the others, and none had any communication with the cavity of the thorax. The lowest tumor, on the left side, contained, besides the serum, which was common to all, several clots, some loose and some attached by bands of fibrin to the walls of the cyst, which were thrown into curious folds, so as to give to the whole an appearance much resembling that of the heart with its musculi pectinati. The other sacs presented a similar surface, but in a less degree.

The larynx and trachea were now opened, when the most important lesion was at once revealed. The mucous membrane of the larynx was faintly reddened, and the rima glottidis almost closed by effusion into the sub-mucous tissue. The child died then of œdema glottidis, brought on, I have no doubt, by the irritating pressure of the distended sacs. This consequence of external tumours of the neck, though rare, is not, I think, wholly unprecedented, as aneurisms have sometimes given rise to it. The viscera, and even the thyroid gland, were perfectly healthy, and no other lesion of much importance was observed.* S. W. M.

Case 2.—During the winter course of 1845–6, Mr. A. G., a farmer, aged 45 years, and in excellent general health, presented

* This case closely resembles one of those reported by Mr. Cesar Hawkins, and I am indebted to my intelligent young friend, Dr. S. Weir Mitchell, for the excellent report of its progress and result. T. D. M.

himself at the " Clinic " for the purpose of having an immense tumour removed from his neck. It appeared that two years before he had suffered from a severe attack of acute inflammation of the throat, which gradually subsided, leaving an induration the size of a marble, near the parotid space. For several years this swelling remained indolent, but at length, without any manifest cause, it began to increase, and gradually attained its present bulk. The tumour occupied the *left* side, and extended from the ear down upon the chest. (See fig. 2.) It also involved the larynx and trachea, and passed across the neck behind. Its surface was perfectly smooth, hard, and of the natural color of the integument. When stricken, a sense of oscillation, rather than fluctuation, was developed. *There was no pulsation at any point*, nor was there much suffering. Occasionally, a sharp lancinating pain would occur; but the chief inconvenience was referable to the *weight* of the mass.

Fig. 2.

After a careful examination, I arrived at the conclusion that it was a cyst, and to verify the diagnosis, introduced a small grooved needle. On withdrawing the instrument, a few drops of a dark chocolate colored fluid escaped, which indicated at once the nature of the tumour.

As the walls of the cyst were somewhat dense, I had no hope of curing the patient radically by any other method than the "seton," but as he objected to this, and begged for a *palliative* operation only, I used the trocar, introducing the instrument at the most dependant part of the tumour, and as the fluid escaped, *scratched* the surface of the cyst with its point. The object of this measure was to excite, if possible, sufficient inflammation to cause the effusion of plasma, and the consequent obliteration of the sac. The fluid, *forty-four ounces in quantity*, and of a dark, chocolate color, having been entirely evacuated, a firm compress and roller were applied over the now flaccid sac, and the patient ordered to remain at rest. His diet was also restricted, and directions given to have his bowels kept in a lax condition for a few days.

It is needless to follow the case through a simple course of treatment, which occupied some two weeks; suffice it to say, that at the expiration of this period, marked by the development of no bad symptoms, the patient returned to the country perfectly free from every vestige of his tumour.

This relief continued for *several months*, when, as I understand, the fluid again accumulated, and was drawn off by some surgeon in his neighborhood.

This case illustrates the benefit to be derived from simply drawing off the fluid from an hydrocele, but it is the duty of the surgeon in similar ones, to state candidly to the patient that from this measure alone a *radical* cure cannot usually be expected. If satisfied to submit to repeated operations, he has certainly the right to do so. *In young persons, and in recent cases*, occasionally, frequent tappings may prove sufficient to accomplish a perfect and thorough cure.

Case 3.—In the fall of 1847, a woman named Jane Gordon, aged 35, of good health, and the mother of several children, came into the institution for the purpose of having a tumour removed from the *left* side of her neck. It occupied the parotid space, extended from the lobe of the ear nearly to the clavicle, and equalled in general bulk a large sized goose egg. It had existed for some five or six years, and could not be traced to any exciting cause. Its growth was gradual, and unaccompanied by pain. Like most tumours of this class, it was smooth, elastic, colorless, obscurely fluctuating, and more or less moveable. Having explained to the patient the nature of her complaint, and also the different methods of treatment, she decided to submit to the *radical cure.*

For the accomplishment of this end, I introduced a *seton,* (by a method hereafter to be described,) and after the escape of the fluid, dressed the wound with a poultice and roller. A general antiphlogistic regimen was ordered, and continued during eight weeks. At the expiration of this period the patient was discharged, cured; and it may be well to state, that no bad symptom of any kind showed itself pending the entire treatment of the case.

The fluid drawn off was straw colored, and contained crystals of cholesterine. It also presented all the traces of an albuminous solution.

Case 4.—In the spring of 1848, I was requested by Mr. Young, residing in Dyllwin street above Noble, to examine a tumour about the size of a small walnut, situated just below the lobe of the *left* ear, which had existed for some time, and was now increasing with considerable rapidity. I found it smooth, elastic, pale in color, painless, firmly attached to the parts in its vicinity, and fluctuating obscurely. The youth, and excellent general health of the patient, led me at once to the conclusion that nothing malignant could be suspected, and the sense of fluctuation indicated that the tumour contained fluid. The diagnosis was, therefore, sufficiently clear, and I advised the patient to submit to the removal of the disease.

Accordingly, in the presence of my class, I *dissected out the cyst*, which contained a dark colored fluid. The wound was closed by a suture, and the cold water dressing applied. No untoward symptoms supervened, and a radical cure was accomplished in the course of a few days.

### REMARKS.

*Definition and History.*—The term "Hydrocele," so generally employed to designate collections of serum in the genital organs of both sexes, was first applied by Professor Maunoir, of Geneva, to tumours containing a fluid, but located in the neck. In the year 1815, a memoir in relation to the subject was submitted by Professor M. to the Royal Institute of France, and afterwards to the Academy of Natural Sciences. By the latter body, Baron Percy was selected to report upon its merits; and strange as it may seem, this distinguished savant not only denied the claims of Prof. M. to the discovery of a new disease, but declared that all the cases of the Professor were nothing more than "*softened bronchoceles*," a class of tumours described or mentioned by many of the older surgeons, especially Celsus, Albucasis, Helwig, Heister, Petit, Plouquet, Louis, Tenon and Pelletan. Others, and among them Foderé, have adopted the same view of the nature of the complaint, but as we shall have occasion to show, without any foundation. That the affection has existed as long as any other disease to which the human frame is liable, there cannot be a doubt, but it must also be confessed that up to the period of the publication of the essay of Prof. Maunoir, its true character had been overlooked, and hence to Prof. M. belongs all the credit of having first pointed out the essential peculiarities of this interesting malady. Within a few years, numerous examples of " Hydrocele of the Neck " have occurred in the practice of different surgeons, and valuable details of cases have been furnished by Dupuytren, Bransby Cooper, O'Bierne, C. Hawkins, and others, all of whom unite in defining the disease in question to be "a tumour or tumours filled with a serous fluid, and occupying *any portion* of the neck,"—thus confirming the view of Prof. Maunoir, who declares it to be " simply a collection of serous or lymphatic fluid."

Causes.—Two varieties of the complaint in question are met with. The first is *congenital*, the second results from the operation of some cause, acting after birth, and may be termed the *accidental* or *acquired.*

It is exceedingly difficult to assign any proximate cause for the congenital variety, and in the attempt to do so, the whole subject of the " development of cysts " is at once called up; and did the occasion permit, the investigation of this interesting point in pathology would prove to you no less interesting than instructive. That there exists, in the laxity of tissues and weakness of vessels of this region, in the fœtus, a *predisposing* cause of effusions of serum, is sufficiently obvious; but what determines *positively* and *directly* the separation of this fluid from the blood, is beyond our ken. Once separated, its collection in sacs or cysts is easily understood.

In that form of the complaint arising after birth, various causes operate in its development. For example: any agent capable of exciting inflammation in the cellular tissue of the neck, may produce an hydrocele, particularly if the diathesis of the individual attacked be such as to favor the separation from the blood of *serum*

rather than *lymph.* It is also possible, as Vidal suggests, for the tumour to originate in "*deposites of blood itself, encysted, and gradually transformed into a serous fluid.*" In other cases the sac appears to be a mere development of a mucous bursa, an opinion expressed by Boyer, in speaking of those cysts which are formed in the space between the thyroid cartilage and os hyoides.

Location of the Tumour.—With the exception of the congenital cases, in which the tumours occupied both sides of the throat, observation, so far, goes to prove that the *left* side is the most common seat of the affection. The swelling frequently commences in the *lower part* of the neck, just above the collar bone, and gradually involves neighbouring portions of this region; but it also originates in other places, particularly the space covering the parotid gland. In determining our diagnosis, the *usual* location of the hydrocele should be borne in mind.

Symptoms.—The symptoms in this disease must depend upon the size, location, variety of cyst, and the nature of its contents. Usually the tumour is smooth upon the surface, of the natural color of the integument, fluctuates, and is *free from all pulsation, unless placed directly over a large artery, or partially emptied.* It is important to recollect the last circumstance, as it offers one of our most valuable diagnostic signs in establishing the true character of the tumour. In congenital cases, or in thin persons, the sac is sometimes almost diaphanous, but from the nature of the fluid contained, is rarely transparent when examined by transmitted light. Unless some important organ, as the larynx, trachea, a large vessel or nerve of the neck, or the œsophagus, is involved, the swelling causes little or no inconvenience, other than that produced by its size and weight. In case No. 2, the patient complained of an occasional darting, sharp pain, of an instant's duration, but with this exception, there was no suffering whatever, except from the weight, (42 ozs.,) which drew his head to one side. When involving the respiratory organs of the neck, the breathing is often very much oppressed, and death, (as in case No. 1,) may ensue from this cause alone. We may also have, as in case No. 2 of Mr. O'Bierne, violent, spasmodic cough. Where the vessels are involved, hemorrhage from the nose, headache, tinnitus aurium, and vertigo, increase the inconvenience and add to the dangers of the case. Deglutition is also sometimes interfered with, when the tumor is located along the œsophagus. In nearly all cases the patient states that the tumour began as a small, firm, distinctly circumscribed insensible lump, growing imperceptibly and slowly, until from some cause calculated to irritate it, or possibly without any such, it suddenly increased in size and grew rapidly. In some instances, the original tumour is traced to pre-existing inflammation, or to the reception of a blow; in others, no possible cause can be assigned.

Such are the ordinary symptoms of a hydrocele of the neck, but there exist modifications of these phenomena, or others of an entirely different character may be added. For example, in case No. 1, the tumour, instead of being one smooth uniform mass, was divided into several distinct sacs, not communicating with each other, and in the centre of the upper sac appeared two *hard and prominent points.* In case No. 2, of Prof. Maunoir, the same fact was observed,—the hard points, corresponding to the location of the thyroid gland. In case No. 3, of Mr. O'Bierne, "the surface projected irregularly at several points, which had a soft, elastic, fluctuating feel; and the skin covering these points was thin, and of a *livid red color;* and when looked at from a distance, the general appearance of the tumour was such, that it might easily be mistaken for '*fungus hæmatodes.*' The patient also complained of 'stinging pains, darting occasionally through the tumour.'"

In the case of Dupuytren, (Révue Médicale) the tumour was "situated between the os hyoides and the thyroid cartilage, so that a finger, passed deep into the mouth, could feel its base behind the tongue." Here "the *breathing was so much obstructed that the patient was in constant dread of suffocation; and his speech became so much affected that he could with difficulty pronounce any long word.*" In Mr. Bransby Cooper's case, the tumour presented "*three distinct sacs, not communicating.*" And in case No. 3, of Mr. C. Hawkins, "*the tumour was composed of a vast number of sacs of different sizes, filled with fluids, differing in color and consistence, and intimately connected with the large vessels of the neck.*" There also existed "*a varicose condition of the vessels of the cheek, resembling that in the vicinity of sanguineous tumours.*"

Diagnosis.—Prof. Maunoir well observes that the disease under consideration must have been often seen without its true character being known. In fact, many of the older surgeons have reported cases of serous tumours of the neck, but always confounded them with *Goitre.* As already mentioned, Baron Percy adopted the same view, and proposed the introduction of the term *Hydrobronchocele*, as better calculated to convey the precise character of the disease, viz., " a solid bronchocele converted into an aqueous tumour."

But all recent observation goes to prove that " hydrocele of the neck" is essentially distinct from " *bronchocele*," under any circumstances. In the first place, *most of the tumours in hydrocele occupy regions of the neck far removed from the thyroid gland.* Reference to the cases reported substantiate this point. In the second—*the disease generally exists independently of any affection of the thyroid gland.* While then, we admit that the thyroid gland is often converted, by softening, into a cyst, it is at the same time evident that " hydrocele of the neck " is a disease distinct and apart from any such affection of that body.

As the tumour may occupy any region of the neck, and from certain peculiarities may want some of the most striking evidences of its precise character, the diagnosis as regards other tumours of the throat becomes sometimes difficult. For example, in the case of Mr. Cooper, the sac was *so deeply situated, and so firmly bound down by fascia*, that the tumour was supposed to be solid, and not until laid bare, was its true nature ascertained. In such cases our only certain method of arriving at an accurate knowledge of the character of the swelling is to introduce carefully, either an *acupuncture or grooved needle, or else a delicate trocar.* The absence of resistance, and the freedom with which its point may be moved in different directions, enables us to detect with the acupuncture needle the existence or non-existence of fluid in the tumour, and where the grooved needle or trocar is employed the escape of the fluid renders the diagnosis at once clear.

*Fatty tumours*, from their yielding a sort of fluctuation, from the absence of pain, from their being smooth on the surface, and from their being usually circumscribed, have been comfounded with hydrocele, but if the least obscurity in the diagnosis exists, *exploration* with the needle or trocar settles the matter immediately.

*Simple glandular* tumours have also been confounded with hydrocele; but the history of the case, the general condition of the patient, the inflammatory pain, the ordinary symptoms of suppuration, present in most of these affections, should render the nature of the complaint sufficiently apparent; but here, as in other cases, *exploration* will always give us correct information.

*Chronic abscess* of the neck, from the fact that in physical characteristics it closely resembles hydrocele, may be mistaken for the latter complaint. I have, in fact, witnessed this error. But the diagnosis is rendered simple by resorting, in all cases of doubt, to the *needle or trocar.*

When occupying the *parotid space*, and firmly bound down by the fascia of this region, it is often difficult to determine the precise character of a *small hydrocele.* It is firm, smooth, natural in color, painless, and circumscribed. Here are all the usual characteristics of *parotid tumour in its commencement.* When of large size, the diagnosis is much more simple. As the prognosis and treatment turn, in such cases, upon an accurate understanding of the nature of the tumor, the surgeon should proceed with great caution; and, if he cannot arrive at his conclusions without, must resort to the needle.

*Hernia bronchalis*, a rare disease, occurring in persons who habitually strain their voices, and which is nothing more than a protrusion of the mucous membrane through the rings of the trachea, or the cartilages of the larynx, has been mistaken for hydrocele; but the *soft*, *elastic* feel of the tumour, the fact of its *disappearing under pressure*, and its *rapid growth from violent exertion*, should enable any one to form a just estimate of the nature of the case.

*Aneurism*, too, has been mistaken for a serous cryst; and this error it appears was committed by even the celebrated Percy,—at all events, he contends, with Albucasis and some of the older writers, that "all tumours composed of *dropsy and goitre*, pulsate synchronously with the action of the heart and subjacent arteries." Now, all observation goes to show that *just the reverse* is the case, unless the cyst be *partially filled* and *placed directly upon the vessel.* In no case have I ever observed any pulsation, and Heister, Maunoir, Lawrence, Delpech, Fleury, Dupuytren, Sabatier, Vidal, O'Bierne, C. Hawkins, B. Cooper, Phillips, in short, all those who have recently reported cases, make a similar statement.

*The absence of pulsation* is, then, a characteristic of hydrocele of the neck, and this fact should ever be borne in mind in making up our opinion in doubtful cases.

O'Bierne has well explained the cause of the absence of pulsation, even where the tumour is in contact with an artery. He observes, " In every stage of the tumour, the sac is so filled, that little or no motion is permitted between the particles composing its fluid, and consequently, according to a received axiom in physics, these particles are incapable of transmitting, in any sensible degree, the impulse communicated to them by the beating of subjacent arteries, until a certain portion of the fluid contents shall have been evacuated."

*Subcutaneous nævus*, a most fearful complaint when it occurs as a congenital defect, and involves, as it often does, the whole front of the neck, has likewise been confounded with hydrocele. I recollect having seen a case of this kind, several years since, with my friend Dr. Janney, in which the deformity was terrible, and from the effects of which the child subsequently died. The *soft pulpy feel of the tumour, its variation in size at different times, the usual purplish color of the skin, and the unusual vascularity of the parts in the vicinity*, are generally sufficient to distinguish the former from the latter affection. However, in the case of Mr. Hawkins, in which a vast number of *small cysts, some half empty*, occupied the anterior region of the throat, the diagnosis was very difficult.

*Malignant tumours* of the neck, might possibly be confounded with hydrocele of the multilocular variety, wherever the skin *becomes red*, (as in the case of Mr. O'Bierne,) *and the tumour painful;* but the *general health of the patient, and the history of the case*, will usually be sufficient to prevent the commission of any serious mistake.

Prognosis.—The prognosis here depends upon circumstances. In nearly every case of congenital hydrocele, the patient has lived but a few months, and every variety of treatment has failed to accomplish more than a mere palliation of symptoms. When developed at a subsequent period, the prospect of relief is much more decided, and, in fact, when properly managed the disease, for the most part, readily yields. When important organs are involved, it will be easily understood that serious inconvenience, and even death, may result from this cause alone. Again, the dangers of the disease are much increased by improper treatment; where, for example, the fluid is allowed to escape into the cellular tissue of the neck, during or after an operation, speedy suffocation, or subsequent serious inflammation might supervene; and if the surgeon attempts, in young subjects especially, to cure the disease by "*stimulating injections*," or by "*filling the sac with lint*," as advocated by some, experience proves that great danger, arising from inflammation, will in nearly every case so treated, sooner or later be developed.

Dissection.—Examination with the knife indicates to us that the sacs here, like those developed in other parts of the body, may present either the *simple*, *multilocular* or *included* variety. In all, we have the peculiar arrangement of tissue which belongs to "*serous cysts*," either natural or acquired. In some cases, more particularly the congenital variety, the sac is so thin as to render the tumour partially diaphanous; in others, the sac is so thick and dense, as to offer a serious obstacle to the radical cure of the complaint. In case No. 2 it was at least four lines in thickness throughout. The effect upon adjacent organs will, of course, depend upon the *size*, *duration*, and *direction* of *growth* of the tumour. According to some, the sac may be composed of a dilated mucous bursa, as when the disease is situated between the os hyoides and thyroid cartilage. Degeneration of the sac is rare, but in one case reported by Fleury, "it was found very hard and resisting, and its interior lined with a fibro-cartilaginous covering!"

Character of the Fluid contained in the Tumors.—The fluid contained in these cysts varies very much in different cases. It has been found limpid, turbid, yellowish, reddish, greenish, and, lastly, coffee or chocolate colored. The last is by far the most common variety, and owes its hue, in all probability, to the presence of the coloring matter of the blood. In consistence it also presents various modifications. Usually it resembles, in this respect, thick cream, but I have known it as thin as water, and again so thick as scarcely to flow through the canula. It is at first inodorous, but changes from exposure to the atmosphere, and becomes very disagreeable, smelling like putrid blood. It is heavier than water, sinking when poured into this fluid, and possesses a sweetish taste like that of pus. In some cases after cooling, the surface is covered with micaceous scales, and in others the microscope detects other ingredients. It is also usually albuminous and coagulates with heat; in some cases it coagulates on exposure to the atmosphere.

I am indebted to Dr. Leidy for the following interesting account of his examination of the fluid drawn from case No. 1.

"The matter contained in the bottle which you sent me for examination, has the appearance and general characters of a coagulum of fibrin from lymph. Chemically it is certainly a proteine compound. Soluble like fibrin or albumen in liquor potassæ, proteine is precipitated from the solution on the addition of an acid. Boiled with hydrochloric acid it gives the characteristic blue color of proteine compounds. With nitric acid and ammonia it becomes yellow, from the formation of xantho-proteate of ammonia. If it coagulated spontaneously, it is undoubtedly fibrin. Microscopically examined, it presents a homogeneous, faintly granular material, with yellowish nuclear bodies diffused through it." (Fig. 3.)

Fig. 3.

In that taken from case No. 2, Dr. H. T. Child found the appearances so well exhibited in fig. 4, and so clearly described in the following note:

*Philadelphia, 3d month 4th,* 1850.

Dear Doctor,—Enclosed I send the drawing of the fluid from the hydrocele of the neck.

Fig. A represents a section of the field of the microscope, with a thin stratum of the fluid upon it. In it we find, 1st, Numerous crystals, of the same form and physical characters of those shown me by my friend, the late Dr. Southwick, who published a report of a case of steatomatous tumors containing crystals, in the Medical Examiner, of this city, vol. vi., p. 283, 1843; they answer the chemical description given by him, being insoluble in water, slowly soluble in alco-

Fig. 4.

hol, and readily dissolved in turpentine. I have seen similar crystals in pathological specimens since that time. They are probably cholesterine.

Fig. B, is a drawing of these magnified about 800 diametres, without any of the surrounding mass. These crystals form slowly upon the glass, and, I presume, do not exist in the fluid before it is evacuated, other than as a crystallizable matter. They were more numerous in portions, the consistency of which was increased by evaporation.

2d. There is a great number of neucleated cells dispersed through every part of the fluid, and, as will be perceived, in several instances these were arranged in a linear form, showing an attempt at organization.

3d. *Epithelial cells.* These varied in different specimens, but were found in all of them. They were evidently thrown off from the walls of the cyst.

4th. *Pus globules.* A few of these were found in several specimens, but their number was inconsiderable.

5th. The remainder of the mass appeared to consist of the *debris* of the various ingredients, some of these presented a caudate appearance.

With sentiments of regard and esteem, I remain thy friend,

H. T. Child.

To Prof. Mütter.

Treatment.—The various methods of treatment to which attention has been mainly directed are the following :—

1st. Excision.

2d. Repeated tappings.

3d. Acupuncture.

4th. Free incision, followed by pressure.

5th. Incision, followed by stimulating applications to the surface of the sac.

6th. Injections.

7th. The seton.

1st. *Excision.*—It must be obvious that this method should be confined to cases in which the tumour is small, circumscribed, and superficial, and *where the existence of a small scar is of not much importance.* Here nothing could answer a better purpose. The patient is speedily and radically cured, and with but little pain or inconvenience. The operation can be performed in one of two ways. Either the skin may be freely divided from without inwards, and the cyst dissected out entire; or else a sharp pointed bistoury passed through the base of the tumour, and made to cut its way out, dividing the cyst in halves, each one of which may be dissected, or drawn out with a pair of forceps. A simple dressing completes the operation.

2d. *Repeated Tappings.*—The operation of repeated tapping may be had recourse to, either as a palliative or radical measure. The objection made to it, that infiltration of the cellular tissue is apt to follow its performance, holds good only when the tumour is but partially emptied. If all the fluid is drawn off, the small opening in the cyst made by the trocar will be closed before any amount of serum can be again secreted. When this is neglected, the effusion, as in one case of Prof. Maunoir, may develope serious consequences. The surgeon should, consequently, never follow the advise of Percy on this point, but carefully take away every drop of fluid that can possibly be removed. Used as a *palliative* measure alone, it answers a good purpose in *congenital hydrocele*, when it would be improper to resort to any severe remedy, and might even accomplish a *radical cure*, by keeping the sac constantly emptied. It may also be employed in *old cases*, to relieve urgent symptoms, or when the patient refuses to submit to any more decided plan of treatment. To increase the chance of a *radical* cure by this operation, we may here, as is often done in hydrocele of the tunica vaginalis, scratch the surface of the sac with the point of the trocar before the instrument is withdrawn. In all cases it is well, after closing the external wound, to make moderate and well directed pressure upon the sac, so as to favor agglutination of its walls.

3d. *Acupuncture.*—Reasoning from analogy, it has been supposed that, by establishing a "*new action*," in the secreting surface of the cyst, a complete arrestation of the secretion might be accomplished. With this view I tried *acupuncture*, in two cases, and found here, as in ordinary hydrocele, that the tumour, for a few days diminished in size, but the improvement was never permanent, the secretion again forming, and the tumour becoming as tense as before. This measure, therefore, I consider of no practical value.

4th. *Free incision.*—Unquestionably the *shortest* plan of curing one of these tumours, if superficial, of the simple variety, and too large for complete removal, is that carried into execution by Heister, Delpech and Lawrence, viz., "*free incision of the sac, followed by moderate compression.*" The operation is soon over, and has hitherto proved successful, but there is one serious objection to its employment, especially in females. It *must of necessity leave a long and unsightly cicatrix on the neck.* Other measures should, therefore, be preferred, *unless the scar is of no moment to the patient.* In such cases it may be resorted to with confidence. As it is sometimes followed by severe inflammation, it should not be performed upon young persons; nor should it be attempted in tumours deeply seated.

5th. From the fact that *simple incision* fails in some cases to accomplish a *radical* cure, attempts have been made by different Surgeons to render the operation more certain, by rapidly converting the *secreting surface* of the sac into a *granulating* one. For the accomplishment of this end, various agents have been employed. Dupuytren introduced pieces of lint down to the bottom of the wound, and allowed them to remain until suppuration was fully established. Hawkins painted the surface over with a solution of the iodide of potassium and iodine; or tinct. of camphor, or a mixture composed of muriate of ammonia, vinegar and alcohol. Others have used port wine, sulphate of copper, &c. In short nearly all the usual measures for changing a serous into a granulating surface, have from time to time been had recourse to.

The operation, however, is one that should be employed with caution; not only must it develope severe *inflammation* at the time, but if successful, the cicatrix never fails to be irregular and of great extent. It is also a tedious method.

6th. *Injections.*—To avoid the scar made by incision, and to accomplish obliteration of the sac at the same time, the operation "*by injection,*" as in ordinary cases of hydrocele, early presented itself to the mind of Prof. Maunoir. He accordingly made the attempt, but found the operation not only of no avail, but when any agent sufficiently powerful to make any impression upon the dense cyst was employed, fraught with danger. To quote his own words,—" An injection," he observes, " which is not very stimulating will effect nothing, or almost nothing, on a very thick, and, in general, an old cyst. If a very active injection be employed, it will cause great pain, and give rise to very alarming spasmodic symptoms. Moreover, I have to observe, that sometimes enlargement of the *thyroid gland* complicates the treatment. In that case, the object is not merely to produce adhesion of the walls of the sac; it will be necessary to employ a mode of cure by which we may succeed at the same time in resolving this gland, when it projects into the tumour, as I have seen in two patients." The cure by " injection," then, is one now almost abandoned.

7th. *The seton.*—The remedy in which most confidence is to be placed in cases where *extirpation*, *repeated tapping*, or *incision followed by pressure*, should not, from the nature of the tumour, be attempted, or where, if put in practice, have failed, is the *seton.* This measure was introduced by Prof. Maunoir, and most modern Surgeons are disposed to agree with him in the estimate he makes of its value. That it is inapplicable to cases of multilocular cyst, unless several setons are used, and also to those occurring at birth, will be readily acknowledged; but nothing promises as *much* when the cyst is simple and of long standing.

The best mode of passing the seton is that proposed by Mr. O'Bierne, as it obviates the hazard of infiltration; an accident very likely to arise when the threads are passed through openings made with a trocar. His plan consists in first making " a *free incision not involving the sac, at the upper and lower extremities of the tumour, by raising and afterwards dividing a transverse fold of the integuments at each of these points.*" Next " the *sac is opened above, and a long probe, armed with a sufficient number of threads, passed down to the most depending point, against which it is firmly pressed.*" Lastly, " *the sac is cut upon the probe, so as to allow the instrument to pass, and the seton to be introduced.*" After the introduction a light emollient poultice makes the best dressing for a few days, when a cerate cloth and simple roller answers until the seton is removed.

This method of treatment is tedious, weeks sometimes elapsing before the cure is completed, but it has the advantage of being *safe*, *easy of execution*, *but slightly painful*, *certain*, and followed by *little or no scar.* During the time of wearing the seton the patient should be careful to avoid exposure, and must also lead a temperate and regular life.

## Figures and Tables

**Figure f1:**
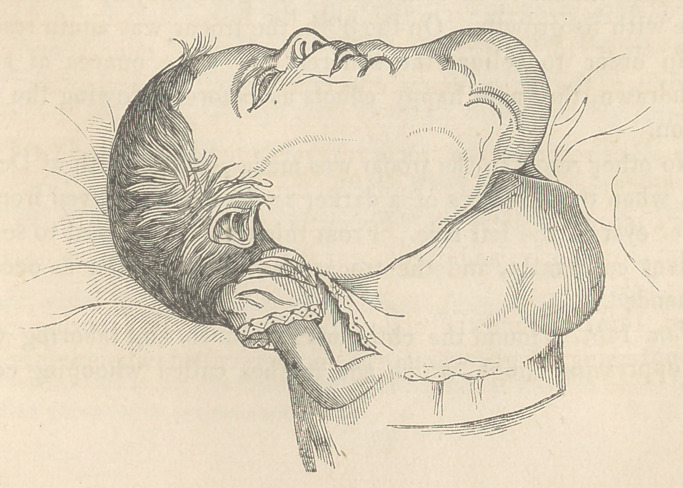


**Fig. 2. f2:**
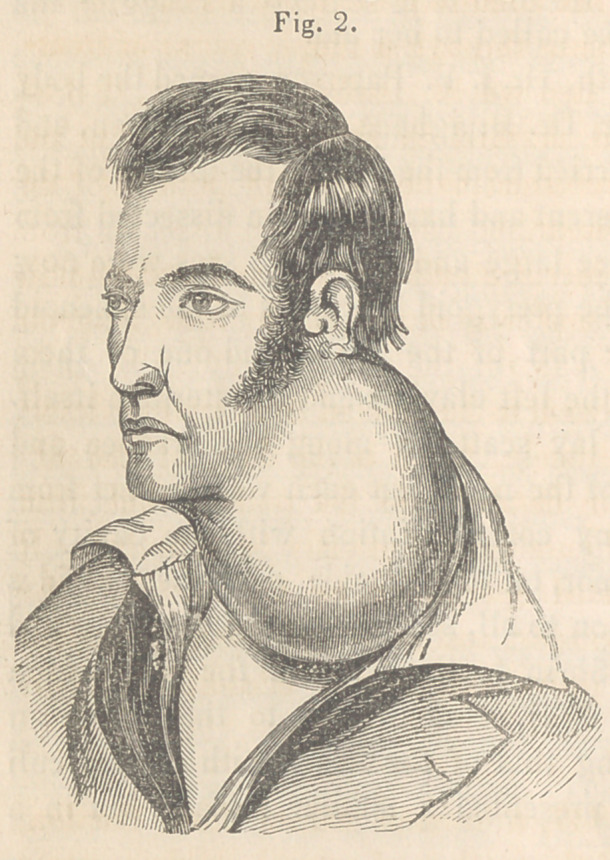


**Fig. 3. f3:**
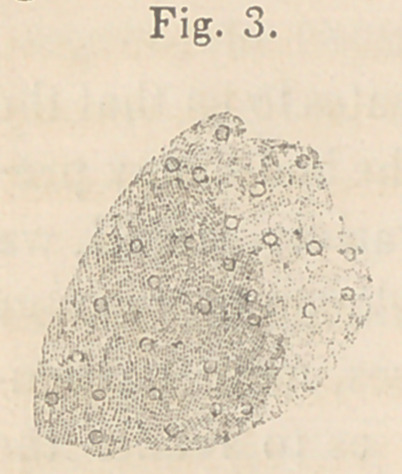


**Fig. 4. f4:**